# Predicting transcriptional responses to heat and drought stress from genomic features using a machine learning approach in rice

**DOI:** 10.3389/fpls.2023.1212073

**Published:** 2023-07-17

**Authors:** Dajo Smet, Helder Opdebeeck, Klaas Vandepoele

**Affiliations:** ^1^ Department of Plant Biotechnology and Bioinformatics, Ghent University, Ghent, Belgium; ^2^ Center for Plant Systems Biology, Vlaams Instituut voor Biotechnologie (VIB), Ghent, Belgium; ^3^ Bioinformatics Institute Ghent, Ghent University, Ghent, Belgium

**Keywords:** rice, regulatory elements, regulation of heat stress, regulation of drought stress, machine learning interpretation

## Abstract

Plants have evolved various mechanisms to adapt to adverse environmental stresses, such as the modulation of gene expression. Expression of stress-responsive genes is controlled by specific regulators, including transcription factors (TFs), that bind to sequence-specific binding sites, representing key components of cis-regulatory elements and regulatory networks. Our understanding of the underlying regulatory code remains, however, incomplete. Recent studies have shown that, by training machine learning (ML) algorithms on genomic sequence features, it is possible to predict which genes will transcriptionally respond to a specific stress. By identifying the most important features for gene expression prediction, these trained ML models allow, in theory, to further elucidate the regulatory code underlying the transcriptional response to abiotic stress. Here, we trained random forest ML models to predict gene expression in rice (*Oryza sativa*) in response to heat or drought stress. Apart from thoroughly assessing model performance and robustness across various input training data, the importance of promoter and gene body sequence features to train ML models was evaluated. The use of enriched promoter oligomers, complementing known TF binding sites, allowed us to gain novel insights in DNA motifs contributing to the stress regulatory code. By comparing genomic feature importance scores for drought and heat stress over time, general and stress-specific genomic features contributing to the performance of the learned models and their temporal variation were identified. This study provides a solid foundation to build and interpret ML models accurately predicting transcriptional responses and enables novel insights in biological sequence features that are important for abiotic stress responses.

## 1 Introduction

Rice is one of the world’s major staple crops. Over 3.5 billion people depend on it for their daily nutritional intake. As the global population is expected to reach ~10 billion people by 2050, we will need to produce more rice on less surface with less input (Wing et al., 2018). However, rice production could be seriously threatened by the aggravating environmental conditions, such as heat, drought and flooding, driven by climate change. To ensure rice production, we are therefore not only in need of plants with a higher yield, but also plants that are more resilient to abiotic stress. A more in-depth understanding of transcriptional signaling cascades in response to environmental stress is therefore needed, as it allows us to engineer rice plants that can better cope with adverse environmental conditions.

As plants have to withstand changing environmental conditions, they have evolved interconnected regulatory mechanisms to sense and respond adequately to adverse conditions (Zhang et al., 2022). Their phenotypic plasticity allows them to survive unpredictable environmental stress. In nature, heat and drought stress often occur together, and untangling the two stresses is not always straightforward (Aslam et al., 2022). At the physiological level, the response to drought and heat stress is regulated by a complex cross-talk between various plant hormones. These reduce respiratory and photosynthetic activity and increase antioxidant response, among others (Wang L. et al., 2020; Li et al., 2021; Iqbal et al., 2022). At the biochemical level, both heat and drought stress cause an increase in the concentration of reactive oxygen species (ROS) (Li et al., 2021; Iqbal et al., 2022). Plants change their antioxidant capacity to maintain cellular redox homeostasis upon sensing stress (Huang et al., 2019; Nadarajah, 2020). At low levels, ROS can function as secondary signals and therefore play a regulatory role in plant stress response (Li et al., 2021; Zhang et al., 2022). In addition to ROS, also cytosolic concentrations of calcium and proline increase in response to heat and drought stress. Similar to ROS, both function as a secondary messenger in signal transduction pathways in response to abiotic stress (Guo et al., 2016; Wilkins K. A. et al., 2016; Iqbal et al., 2019; Takahashi et al., 2020; Zhang et al., 2022).

Environmental stress induced signal transduction triggers genome-wide transcriptional reprogramming, which activates protective mechanisms. Although the physiological effects of stress are extensively studied, there is an incomplete understanding of the regulatory mechanisms involved in the gene expression underlying these effects. An important role is reserved for transcription factors (TFs), which comprise a substantial portion of the protein-coding genes (around 5% for rice, 6% for *Arabidopsis* and 7% for maize)(Jin et al., 2017). They orchestrate gene regulation by sequence-specific binding to TF binding sites (TFBSs) in noncoding regions of the DNA, also known as cis-regulatory elements (CREs), located upstream, in introns, or downstream of the gene body. Together with transcription cofactors and RNA-polymerase II, TFs are key components of gene regulatory networks, which describe the interactions between TFs and the target genes they regulate (Zrimec et al., 2020; Zrimec et al., 2022). Identifying CREs in the noncoding DNA can help to identify functional TFBSs and further unravel the regulatory grammar of abiotic stress response. TFs can also bind DNA cooperatively, hereby enlarging TF functionality through combinatorial control (Lai et al., 2019; Ibarra et al., 2020). The activation of different TFs is triggered by signaling cascades to control the expression of genes that are essential for plant tolerance to drought and heat stress (Guo et al., 2016; Aslam et al., 2022; Hu et al., 2022). Among these TFs, the plant heat stress transcription factors (HSFs) are particularly important for the response to heat stress (Zhang et al., 2022). Other TF families, including NAC, WRKY, bZIP and MYB, are also involved in the regulation of the expression of heat-responsive genes (Zhao et al., 2020). In response to drought stress, TF families such as MYB, WRKY, AP2/ERF and NAC play an important role in the downstream transcriptional reprogramming (Tang et al., 2019; Aslam et al., 2022). Gene expression regulation is, however, not restricted to noncoding regions. Recently, it has been shown that coding regions should be part of the gene regulatory structure (Zrimec et al., 2020; Zrimec et al., 2022). The complete gene sequence is in fact highly predictive of the level of expression (Washburn et al., 2019; Zrimec et al., 2020; Meng et al., 2021).

Elucidating gene expression properties will affect our understanding of plant physiology and help improve crop productivity (Zrimec et al., 2020; Jores et al., 2021). Predicting gene expression patterns is one of many biological applications enabled by advances in supervised machine learning (ML) (Washburn et al., 2019; Zrimec et al., 2020; Meng et al., 2021; Moore et al., 2022; Zrimec et al., 2022). Broadly, it can be divided into classical ML and representation learning. Although representation learning has recently gained a lot of attention with the applications of deep learning, classical ML still has some advantages including ease of configuration, lower computational demands, applicability on smaller datasets and, importantly, more straightforward model interpretation. Classical ML has been successfully applied in recent years to study transcriptional regulation of abiotic stress in plants. Azodi and collaborators used an ML approach to classify gene expression in response to simultaneous heat and drought stress by training models on a combination of putative CREs (pCREs), TFBSs, chromatin accessibility, histone modifications, sequence conservation and other features (Azodi et al., 2020). Zhou and collaborators predicted heat and cold stress responsive genes in maize based on promoter features and epigenetic marks to assess variable or consistent expression response across maize genotypes (Zhou et al., 2022). Other studies have shown that models trained on regulatory elements or mono- and dinucleotide frequencies of both coding and noncoding DNA can predict whether a gene is differentially expressed or nonresponsive in response to cold stress and wounding (Meng et al., 2021; Moore et al., 2022). In rice, Kakei and collaborators showed that pCREs and known TFBSs can be used to train a model that could help unravel the regulatory grammar of iron response (Kakei et al., 2021).

Here, we apply classical ML to gain insight in the regulation of the heat and drought stress response in rice. With this study, we tackle some methodical and data-specific questions concerning ML-based prediction of gene expression, uncovered by previous studies. These include: 1) How does the selection of differentially expressed genes influence the prediction performance? 2) Does a long promoter contain more valuable information for predicting gene expression compared to a shorter one? 3) What k-mer finding strategy reveals the most useful pCREs for predicting the transcriptional response to heat or drought? Taking into account the newly acquired knowledge from addressing these research questions, both coding and noncoding features were used to train ML models that can successfully discern upregulated from nonresponsive genes. To gain novel insights in the regulation of gene expression in response to abiotic stress, interpretability of ML models is of major importance. The most important features for gene expression prediction were identified to help decipher the temporal regulation of the transcriptional response to these increasingly relevant abiotic stresses.

## 2 Materials and methods

### 2.1 Transcriptome data collection and differential expression analysis

Our study is based on the heat and drought RNA-sequencing (RNA-seq) data published by Luo and collaborators (Luo et al., 2019). Each stress was applied to two-week-old seedlings for various periods of time (0h, 3h, 6h, 12h, 24h, 36h and 48h). For heat stress, plants were incubated at 45°C, whereas drought stress was simulated by placing the seedlings into a polyethylene glycol 6000 (PEG-6000) solution. Bulk RNA was extracted from both stem and leaf tissues. Paired-end reads were downloaded from the SRA archive (SRP190858). General feature format (gff) and fasta files were downloaded from Ensembl Plants (Cunningham et al., 2022). We used *Oryza sativa*, ssp. japonica annotation version 1.0.51 (Oryza_sativa.IRGSP-1.0.51.gff3.gz) and removed overlapping genes. Oryza_sativa.IRGSP-1.0.dna_sm.toplevel.fa.gz was used as the reference genome file. RNA-seq reads were clipped with Trimmomatic (Bolger et al., 2014) (settings: ILLUMINACLIP: TruSeq2-PE.fa:2:30:10:2:True SLIDINGWINDOW:4:15). These reads were used for read mapping with Salmon v1.3.0 (Patro et al., 2017) using a decoy database. The resulting count matrices were used for differential expression analysis with EdgeR where time point 0h was used as a control. Upregulated genes were defined based on a log2 fold change (log2FC) >= 1 at any time point, while the False Discovery Rate (FDR) was controlled at 0.05 using glmTREAT (Chen et al., 2016). Genes were considered nonresponsive if the FDR-adjusted p-value was > 0.05 and log2FC was between 1 and -1.

### 2.2 Gene family clustering

Genes were clustered into orthogroups, which we use as a proxy for gene families, using Orthofinder (version 2.3.3) (Emms and Kelly, 2019). Orthogroups of protein-coding genes were inferred across multiple species (*Brachypodium distachyon*, *Sorghum bicolor*, *Zea mays*, *Oryza sativa*, *Setaria italica* and *Triticum aestivum*). One fasta file per species, containing amino acid sequences for the proteins, was downloaded and used to run Orthofinder with the default settings. All fasta files were downloaded from Ensembl plants, release 51, except for *Zea mays*, which was downloaded from PLAZA Monocots 5.0 (Van Bel et al., 2022) (proteome.selected_transcript.zma.fasta.gz from https://ftp.psb.ugent.be/pub/plaza/plaza_public_monocots_05/Fasta/). In cases where a gene could not be assigned to any existing gene family, it was considered to be part of a singleton gene family.

### 2.3 Binning and undersampling of upregulated and nonresponsive genes for class balancing

Genes were sorted in ascending order based on their average Transcripts per Million (TPM) (as measure for baseline expression) and divided into 5 equally-sized bins. The preferred number of bins was based on the minimal loss of upregulated genes. Within each bin, the genes of the most abundant class were undersampled to equal the number of genes of the less abundant class. To do so, genes were sorted based on log2FC within each bin. In case of upregulated genes, those with the highest log2FC were kept in the bin, whereas for nonresponsive genes those with a log2FC closest to zero were retained.

### 2.4 Building the genomic feature space

#### 2.4.1 Promoter definition

Because apart from the region upstream of the transcription start site (TSS) also the 5’UTR is involved in gene expression regulation (Sharon et al., 2012; Redden and Alper, 2015; Srivastava et al., 2018) and because our goal is to further unravel the regulatory grammar, we wanted our promoter definition to include part of the 5’UTR. UTR annotation is incomplete for the rice genome, and UTR length varies across genes. The 5’UTR length ranges from 1 to 7147 for all genes in the rice gff3, with a median of 104 bp. To ensure equal promoter lengths and comparability across genes, we defined the distal promoter as 900 bp upstream of the TSS and, based on the median 5’UTR length in the rice gff3, the first 100 bp downstream of the TSS, and the proximal promoter as 200 bp upstream of the TSS and the first 100 bp downstream of the TSS.

#### 2.4.2 Nucleotide and dinucleotide content

The nucleotide and dinucleotide content of rice genes was quantified using the code published by Meng and collaborators (Meng et al., 2021). The (di)nucleotide content was obtained for 5 genomic regions: upstream of the 5’UTR (900 bp or 200 bp upstream of the TSS, depending on the used promoter definition), the estimated 5’UTR (100 bp based on calculated median 5’UTR length), the CDS, intron and the estimated 3’UTR (250 bp based on calculated median 3’UTR length). The nucleotide and dinucleotide content of the aforementioned genomic regions were used as features for model training.

#### 2.4.3 Known transcription factor binding sites

TF motifs, modeled as position weight matrices (PWMs), were collected from JASPAR 2020 (Fornes et al., 2020) and CIS-BP version 2.00 (Weirauch et al., 2014). Pairwise comparison of PWMs was used to remove duplicates using the Regulatory Sequence Analysis Tools (RSAT) (Castro-Mondragon et al., 2017) program “compare-matrices” with the normalized correlation (Ncorr) as similarity metric. PWMs with an Ncorr of 1 were considered duplicates. Cluster-buster (CB), compiled on Sep 22, 2017 (Frith et al., 2003) and Find Individual Motif Occurrences (FIMO version 4.11.4) (Grant et al., 2011) were used to map the motifs on the noncoding genome. Before the motif mapping with CB, the PWMs were scaled to 100. The command line options used for each tool were “fimo -o $output $PWMfile $seqFile” and “cbust-linux $PWMfile $seqFile -c 0 -f 1”. Following Kulkarni and collaborators (Kulkarni et al., 2019), the top motif matches of each motif were used, with a maximum of 5000 motif matches for CB and a maximum of 9000 motif matches for FIMO.

#### 2.4.4 Putative cis-regulatory elements

To identify overrepresented pCREs in the (distal or proximal) promoter of rice genes, three different k-mer finding approaches were compared: 1) RSAT oligo-analysis and 2) RSAT oligo-diff (ROD) of the RSAT motif discovery suite (Defrance et al., 2008; Santana-Garcia et al., 2022, and 3) the progressive k-mer growing (PG) strategy developed and adopted by (Moore et al., 2022). To prevent leakage from the test set to the train set (Whalen et al., 2022), test set genes were excluded from any k-mer enrichment analysis. To obtain biologically relevant pCREs, particularly when working with gene subsets (see later), significantly enriched k-mers in the upregulated train set genes compared to all nonresponsive genes were identified. Using ROA, a custom background model was created for 6, 7 and 8-mers based on the promoter sequences of all nonresponsive genes. Using ROD, the upregulated genes of the train set were directly compared to the sequences of all nonresponsive genes. Promoter sequences were purged in ROA and ROD prior to k-mer enrichment analysis. Both tools were used to detect enriched 6, 7 and 8-mers separately (no growing procedure). The lower occurrence threshold was set to 3. K-mers with an occurrence signal > 0 and p-value < 0.01 were considered significantly enriched. Using PG (Moore et al., 2022), all possible k-mers of length 6 and longer were identified and tested for significant enrichment in the promoter sequences of train set upregulated genes compared to all nonresponsive genes. A p-value cutoff of p < 0.01 for the Fisher’s exact test and FDR correction was used to determine significantly enriched k-mers. Starting from 6-mers, oligomers were progressively grown until the p-value could no longer be lowered by further extending a given k-mer. Finally, the enriched k-mers resulting from the three different approaches were mapped to the promoter sequences of train and test set genes using the RSAT DNA-pattern tool (Santana-Garcia et al., 2022). The presence or absence of the enriched pCREs in the promoter of a gene were used as features for model training.

### 2.5 Enrichment analysis of TFBSs and Gene Ontology

TFBS and Gene Ontology (GO) enrichment were computed using the hypergeometric distribution, with a gene-class file and a gene-TFBS or gene-GO term file as input (Kulkarni et al., 2018). The gene-class file was based on the differential expression analysis, the chosen subset and chosen definition of nonresponsive genes. The gene-TFBS feature file was built using bedtools intersect (Quinlan and Hall, 2010) on the motif mapping output and the coordinates of the chosen promoter (proximal or distal), discarding unexpressed genes. The gene-GO term file was built as follows: functional gene annotations were downloaded from PLAZA monocots 5.0 (Van Bel et al., 2022). First, the annotations were extended with their parental terms, where necessary. Then, all terms were filtered for “biological process” GO terms. For each enriched TFBS or GO term, the q-value of enrichment was determined using Benjamini–Hochberg correction for multiple hypotheses testing. TFBSs and GO terms were clustered based on the q-value of the fold enrichment, defined as –log10(q–value)^3^, in upregulated compared to nonresponsive genes. The clustering algorithm selected in the seaborn clustermap library (Waskom, 2021) is “average” hierarchical clustering using the metric “Euclidean distance” between the plotted enrichment vector of each motif.

### 2.6 Gene-family-guided train-test split for the complete gene set and subsets

Following balancing (binning and undersampling) of the number of upregulated and nonresponsive genes, the data was divided into train and test sets for supervised ML training and testing. A gene-family-guided approach to train-test partitioning was employed (Washburn et al., 2019). No gene family was allowed to occur in both the train and the test set. The train and test set represent 80% and 20% of the total genes (sum of upregulated and nonresponsive genes), respectively. To ensure a representative test set at each split, the distributions of bins, classes, log2FC and average TPM were compared between the train and test set. A split was not withheld when a significant difference was detected (p < 0.05). Furthermore, for each train-test split, five duplicate RF models (Breiman, 2001) were trained to classify train and test set genes based on the features used for predicting gene expression in response to abiotic stress (see below). The area under the Receiver Operating Characteristic (ROC) curve (AU-ROC), which plots the true positive rate (TPR) as a function of the false positive rate (FPR), was used to assess whether an RF classifier is able to discern the train from the test set. If the median AU-ROC was around 0.5 a split was withheld.

### 2.7 Random forest classifier training, validation, testing and evaluation

**Figure 1:**
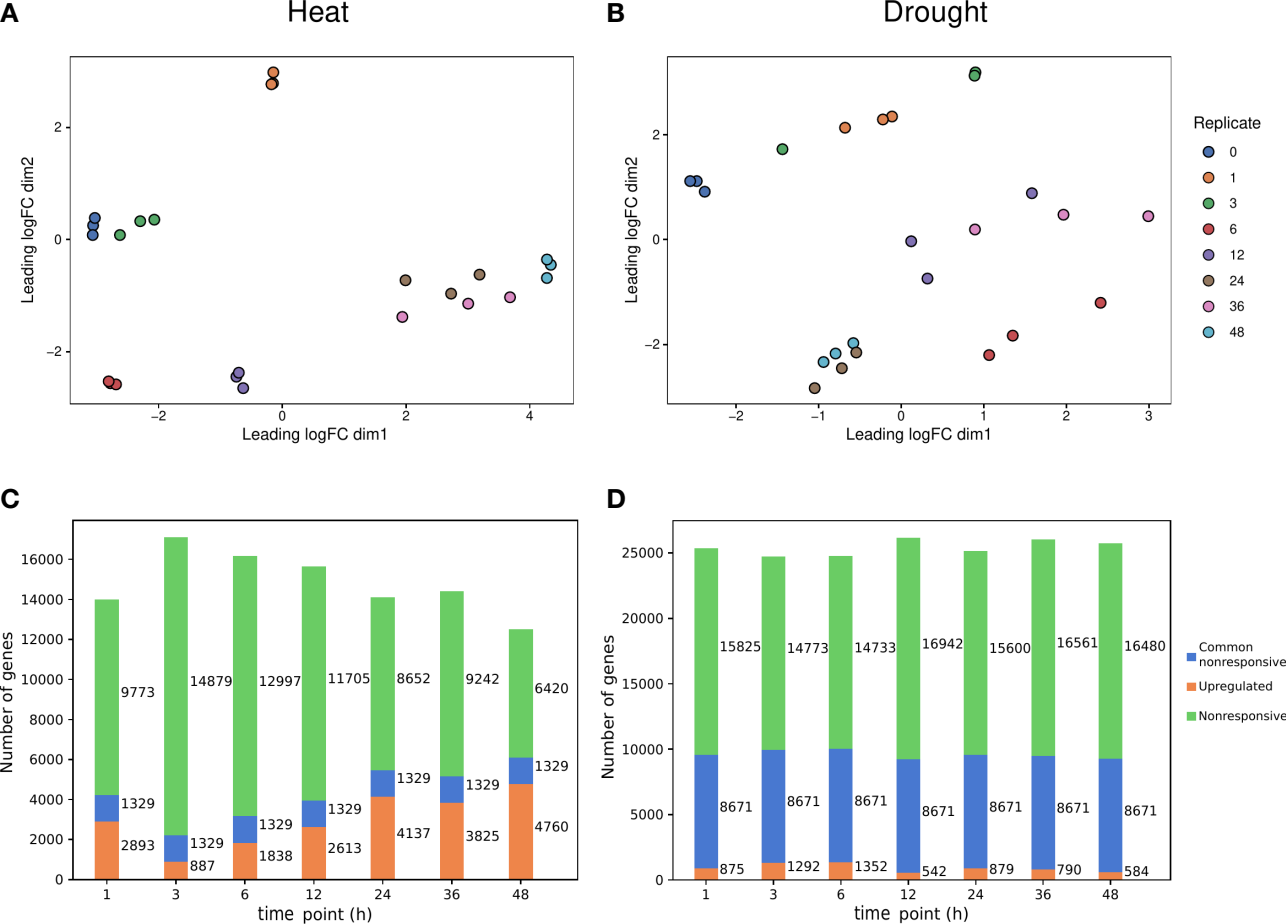
Transcriptional responses induced by heat and drought stress. **(A, B)** Multidimensional Scaling (MDS) plot for heat and drought stress expression data, respectively. **(C, D)** Number of significantly upregulated (orange) and nonresponsive (green) for each time point, and common nonresponsive genes (blue) across time points under heat and drought stress, respectively.

**Figure 2:**
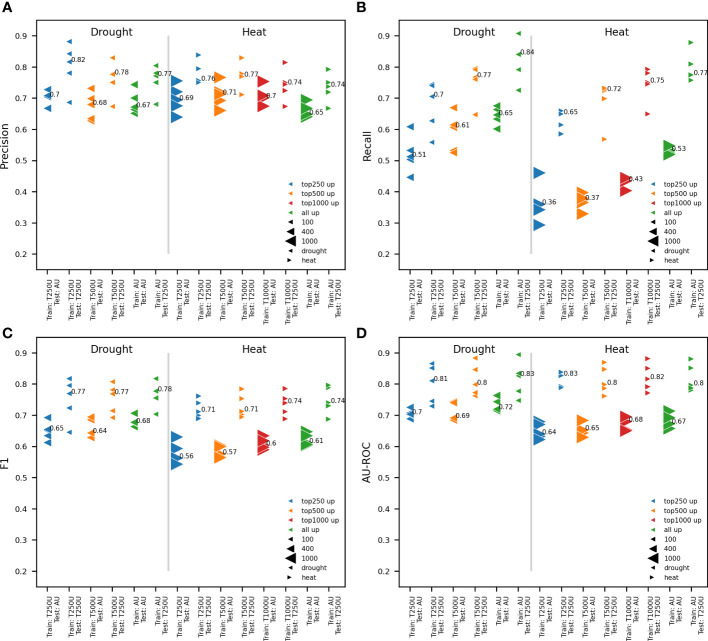
Impact of the quantity of upregulated genes on model performance. Comparison between RF models trained on all available upregulated genes and the 250, 500 and 1000 most strongly upregulated genes (AU, T250U, T500U and T1000U, respectively) based on log2FC. pCREs, identified using RSAT oligo-diff, and TFBSs mapped to the proximal promoter were used for model training. Two test sets are used for testing the performance of trained models: the test set for all upregulated and the 250 most responsive upregulated genes. Results are shown for five independent train-test splits and the median performance is reported. Different model performance metrics are compared: precision **(A)**, recall **(B)**, F1 **(C)** and AU-ROC **(D)**. A different marker is used for heat and drought, while the number of upregulated genes (up) used is shown in a different color. The size of the test set is represented by the size of the marker.

**Figure 3:**
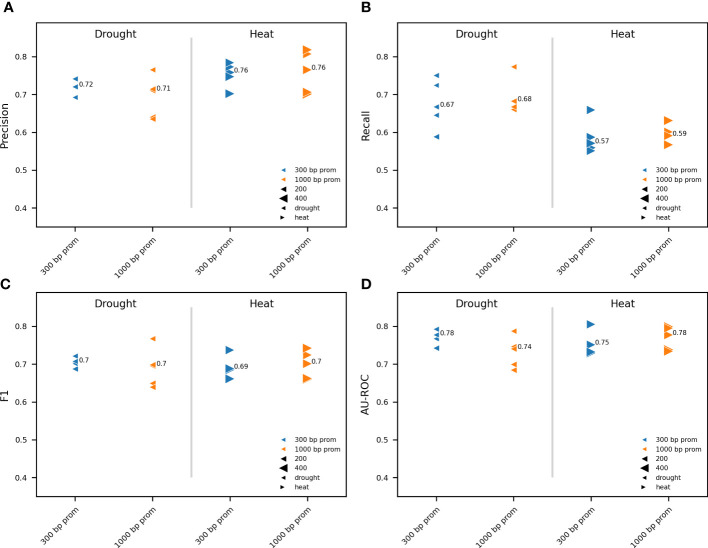
Impact of promoter length on model performance. Comparison between RF models trained on pCREs, identified using RSAT oligo-diff, and TFBSs mapped to the proximal (300 bp) and distal (1000 bp) promoter. For drought and heat, the 500 and 1000 most strongly upregulated genes, respectively, were used for model training. Results are shown for five independent train-test splits. A different marker is used for heat and drought. Promoter lengths are depicted in a different color. The size of the gene subset-specific test set is represented by the size of the marker. Different model performance metrics are compared: precision **(A)**, recall **(B)**, F1 **(C)** and AU-ROC **(D)**. The median performance is indicated.

**Figure 4:**
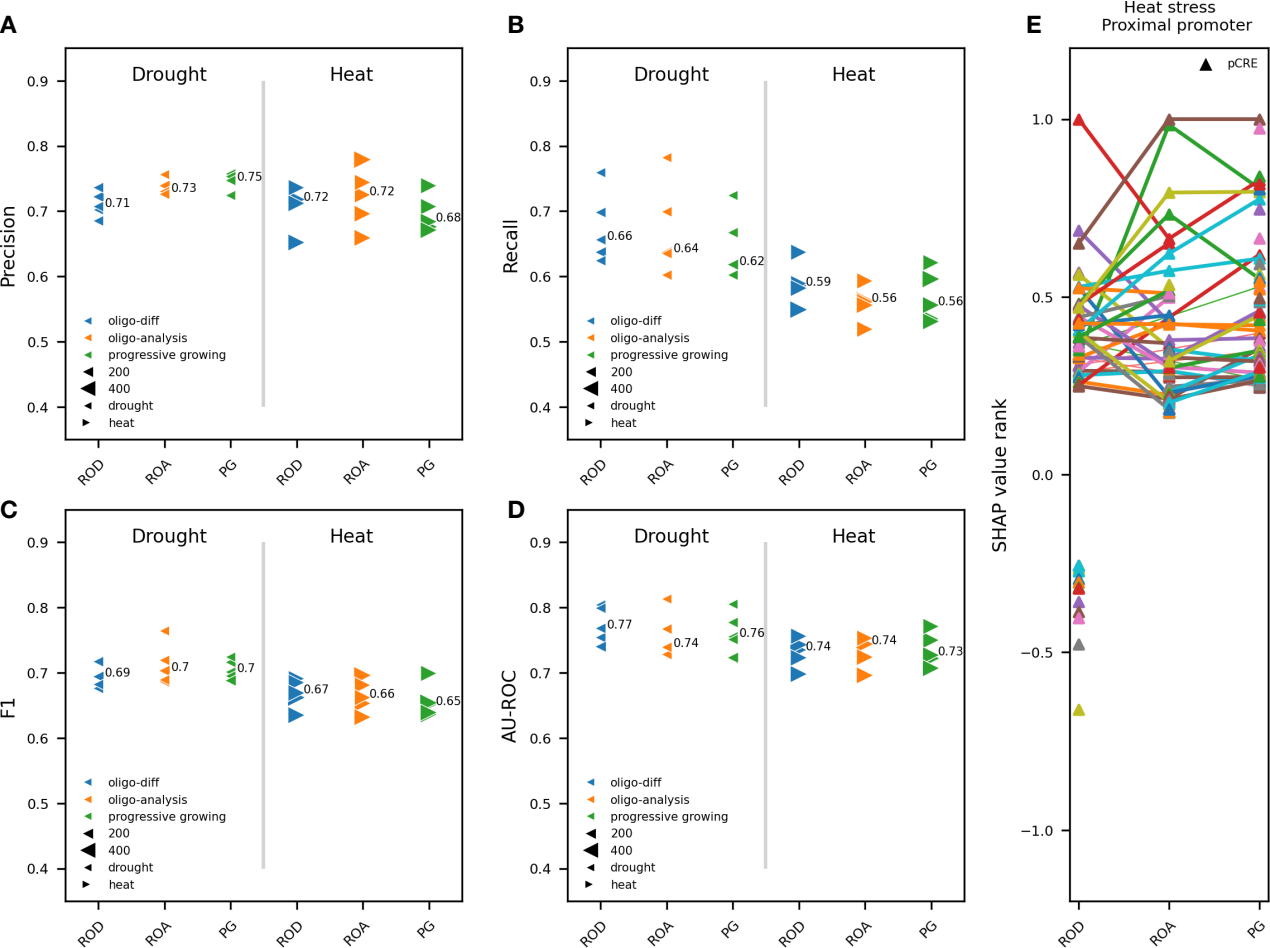
Impact of overrepresented pCREs from different k-mer finding approaches on model performance. Comparison between RF models trained on pCREs identified using a progressive k-mer growing strategy (PG), RSAT oligo-diff (ROD) and RSAT oligo-analysis (ROA), in the proximal promoter of upregulated compared to nonresponsive genes in response to 1h of heat or drought. The 500 and 1000 most strongly upregulated genes for drought and heat, respectively, were used for model training. Different model performance metrics are compared: precision **(A)**, recall **(B)**, F1 **(C)** and AU-ROC **(D)**. Results are shown for five independent train-test splits. A different marker is used for heat and drought. K-mer finding approaches are depicted in a different color. The size of the gene subset-specific test set is represented by the size of the marker. The median performance is indicated. **(E)** The most important pCREs based on SHAP value rank for predicting gene expression in response to heat stress are compared between the three k-mer finding approaches. Common pCREs between different k-mer finding approaches are connected using a line.

Genes belonging to a holdout test set, obtained after each gene-family-guided train-test split (20% of the data), were never used for model training. RF models were trained using the training data (80% of the data) only. A nested cross-validation procedure was implemented to evaluate and compare tuned ML models. The training data was subjected to fivefold cross-validation to train RF models. Within each of the five splits of the cross-validation procedure, fivefold grid search cross-validation was performed over the value ranges of the selected RF hyperparameters (min_samples_leaf = 2, 3, 4; n_estimators = 500, 1000; max_features = sqrt, log2). Relevant hyperparameter value ranges were narrowed down using an exhaustive grid search. The optimal hyperparameters were used to fit a model on all but one fold (four in this case) and the fit model was evaluated on the remaining fold (validation fold). Averaging the outcome over the five splits of the cross-validation procedure provides more reliable results as estimation variance is reduced (Seibold et al., 2018). The best performing model out of the five cross-validation splits (based on F1) was used to predict the class of the holdout test set genes. Various metrics were employed to compare model performances: precision, recall, F1 score, AU-ROC, and area under the precision-recall (PR) curve (AU-PR). The precision is the number of true positives divided by the total number of predicted positives. The recall (also known as ‘true positive rate’’) is the number of true positives divided by the total number of positives. The F1 score (also known as the ‘F-measure’) is the harmonic mean of precision and recall. The PR curve plots precision as a function of recall (Whalen et al., 2022). Also, the confusion matrix and classification report were calculated for the holdout test set. The latter provides important insights in differences in model performance for upregulated and nonresponsive genes. For **Figures 1**–**4**, a model is trained on each of the five representative train test splits using time point specific nonresponsive genes (see above) and the performance metrics (precision, recall, F1 and AU-ROC) are reported for each train-test split.

### 2.8 Feature importance estimation

To determine for each time point the most important features for distinguishing between upregulated and nonresponsive genes, a final model was trained on all available training data. For final model training, the hyperparameter values of the top-performing model, obtained from the previous nested cross-validation procedure, were used. Shapley additive explanation (SHAP) values were used as a measure of feature importance (Arrow et al., 1953; Lundberg and Lee, 2017). Five duplicate models were trained for each time point. Within each duplicate, local SHAP values were calculated for each feature across genes using TreeExplainer (Lundberg et al., 2020). To obtain global SHAP values, the median SHAP value for each feature across genes was first calculated. Subsequently, the median SHAP value across duplicates was computed. Global SHAP values were normalized by scaling them between -1 and 1 using the formula $\frac{SHAPvalue}{max(|SHAPvalue|)}$ . To identify the main features for gene expression prediction, features were sorted in ascending order based on their absolute SHAP value. KneeLocator of the Kneed python module (Satopaa et al., 2011) was used to determine the knee of the absolute SHAP value distribution across features. All features right to the knee were defined as the main features for gene expression prediction, the others were not considered. To compare time points based on most important motifs (pCREs and TFBSs), a hierarchically-clustered heatmap was used. For the top 25 motifs with positive SHAP value, the motif importance (SHAP value rank), enrichment, and similarity was computed. To calculate the motif enrichment, we used the formula:


$$log_{2}(\frac{\mathrm{number of upregulated genes whose promoter contains the motif / total number of upregulated genes}}{\mathrm{number of nonresponsive genes whose promoter contains the motif / total number of nonresponsive genes}}).$$

A positive enrichment is indicative of a motif being present in more upregulated compared to nonresponsive genes. To compute the similarity of pCREs with known TFBSs, PWMs of known rice TFBSs were obtained from CIS-BP (Weirauch et al., 2014) and pCREs were converted to PWM format. Next, the pCREs and known TFBSs were compared with the compare-matrices tool from RSAT (Santana-Garcia et al., 2022) and the Ncorr was computed for each pairwise comparison. All pCREs related to known TFBSs reported below have an Ncorr value of at least 0.4.

## 3 Results

### 3.1 Heat and drought responsive genes and variation in responsiveness across time points

To determine the upregulated and nonresponsive genes for heat and drought and to understand how the transcriptional response for both abiotic stresses varies across time, we used the heat and drought stress response data from a previously published expression dataset (Luo et al., 2019). Two-week-old rice seedlings were subjected to either heat or drought stress over different time intervals (see Materials and Methods). Upregulated and nonresponsive genes were identified at different time points relative to the onset of stress imposition (0h, control). For heat (**Figure 1A**), the majority of time points are clearly separated from the control (0h). The later time points (24, 36 and 48h) are grouped closely together and are distinctly separated from the earlier time points. For drought (**Figure 1B**), the control is clearly distinct from other time points, while for some time points, replicates do not group together as well as others. Time points that are 24 hours apart group together (12 and 36h, 24 and 48h). Of the differentially expressed genes, only upregulated genes were considered here because our goal is to gain further insight into the mechanism of gene expression activation in response to abiotic stress. When studying the regulation of transcriptional responses to abiotic stress, upregulated genes hold more biological significance. Upregulated genes more often signify an active response to stimuli, such as abiotic stress, than downregulated genes. Both abiotic stresses are major drivers of gene expression changes over time, as more than 4700 and 1300 unique genes are upregulated in response to heat or drought, respectively, at some point (**Figures 1C, D**). Hence, heat stress caused more genes to be upregulated across time points compared to drought stress. There is a greater prevalence of nonresponsive genes and a higher number of common nonresponsive genes across time points for drought compared to heat stress. The latter suggests that upon exposure to heat, more genes respond at any of the profiled time points and there is more variation in upregulated genes over time, compared to drought.

### 3.2 Assessing the significance of the quantity of upregulated genes, promoter length and k-mer finding strategy in predicting the transcriptional response to abiotic stress

Various TF families involved in regulating the transcriptional response to heat or drought have been established (Tang et al., 2019; Zhao et al., 2020; Aslam et al., 2022; Hu et al., 2022; Zhang et al., 2022). To further unravel the regulatory grammar of gene expression regulation by TFs in response to heat or drought, we used both known (TFBS) and putative (pCRE) regulatory motifs identified for a specific set of stress-responsive genes. Identifying pCREs important for gene expression prediction in response to abiotic stress can confirm the significance of known TFBSs as well as uncover novel regulatory motifs. Before modeling the temporal response to heat or drought, we first contemplated and addressed diverse technical and data-specific challenges associated with ML gene expression prediction.

Dealing with imbalanced classes is a common challenge in supervised ML on differential expression data. There are considerably more nonresponsive compared to upregulated genes, particularly in response to drought stress (**Figures 1C, D**). To prevent overfitting of ML models on the overabundant nonresponsive genes, a balancing strategy was used. Genes were divided in bins based on average expression to reduce the bias of baseline gene expression (Meng et al., 2021). Within each bin, genes were undersampled to balance the number of upregulated and nonresponsive genes. In addition to the approach used by Meng and collaborators (Meng et al., 2021), we used non-random undersampling to increase the contrast between the upregulated and nonresponsive genes used for training. Only the most responsive and least responsive genes were kept (based on log2FC). Paralogous genes resulting from gene duplication often share highly similar promoters and have similar expression levels. Relatedness of genes between training and test set could lead to unaccounted overfitting on shared gene families when they are randomly assigned to train and test set. To overcome this hurdle, a gene-family-guided train-test split strategy was used, ensuring that a gene family was never represented in both the train and test set (Washburn et al., 2019).

Different ML algorithms can be employed to predict gene expression (random forest classifier, gradient boosting classifier, support vector machines, etc.). Similar to previous studies (Azodi et al., 2020; Meng et al., 2021; Moore et al., 2022), we used RF algorithms (Breiman, 2001) with nested cross-validation to build models for predicting gene expression in response to abiotic stress (see Materials and Methods). RF models cope well with small sample sizes and highly dimensional feature spaces. They are easy to tune compared to other ML algorithms and perform already well with default hyperparameter values (Fernández-Delgado et al., 2014; Probst et al., 2019). Hyperparameter tuning was, nevertheless, used to optimize hyperparameters for RF model training. To gain novel insights in gene expression regulation in response to abiotic stress, interpretability of ML models is as important as their prediction accuracy. We used Shapley, a game theoretic approach, to increase the interpretability of an ML model (Lundberg et al., 2020). The SHAP value, a measure of the contribution of each feature to a correct prediction of the class of each sample (local interpretability), was calculated for each feature in the feature space. One can estimate the global importance of each feature by averaging the local importance for each sample over all training samples (global interpretability). More important features for a correct prediction have a higher global SHAP value (Arrow et al., 1953; Štrumbelj and Kononenko, 2014). As opposed to impurity-based importances (Breiman, 2001; Altmann et al., 2010) used by Moore and collaborators (Moore et al., 2022), SHAP values can be negative. In game-theory, positive SHAP-values are indicative of winning, while negative SHAP-values are indicative of losing. Translated to gene-expression prediction, positive values are indicative of upregulated genes, whereas negative values are indicative of nonresponsive genes. Furthermore, SHAP values are not biased by continuous feature values, when compared to binary feature values. An additional advantage of SHAP values over permutation based importances used by previous studies (Azodi et al., 2020; Meng et al., 2021), is that it can provide insights in whether a high or a low feature value contributes to the prediction of a class.

When the goal is to train a model to discern upregulated from nonresponsive genes, there is a potential trade-off between using more genes and the most informative genes. Working with too many upregulated genes that are not strongly responsive to an abiotic stress could hinder the identification of stress-specific motifs for gene expression prediction. Reversely, using too few genes could decrease overall model performance. To tackle this, the ability to predict gene expression in response to 1h of heat or drought was compared when using all or only the most responsive upregulated genes and an equal number of time point-specific nonresponsive genes. Five representative train-test splits were performed and the train and test sets for the 250, 500 and 1000 upregulated genes with the highest log2FC and the same number of nonresponsive genes with the lowest log2FC, were extracted from these. These gene subsets will be referred to as the top 250, 500 and 1000 upregulated genes. For heat, the 250, 500 and 1000 most upregulated genes were used. For drought, there are less than 1000 upregulated genes at time point 1h (**Figure 1D**). Consequently, only the 250 and 500 most strongly upregulated genes were used. For both heat and drought, RF models were trained on the complete gene set and the different subsets of most responsive upregulated genes. The comparison of model performances is only valid when using a similarly sized test set. Trained models were therefore assessed using both the test set of the 250 most responsive upregulated genes subset, and the test set of the complete gene set (**Figures 2A–D**). The precision was roughly similar for models trained on different subsets of the most responsive upregulated genes and models trained on all available upregulated genes. The median precision varied maximally 0.05 for drought and 0.06 for heat across train sets within test sets (**Figure 2A**). The recall, however, increased when a model was trained on more upregulated genes. The median recall varied maximally 0.14 for drought and 0.17 for heat across train sets within test sets (**Figure 2B**). The increase in recall suggests that there are less false negatives (FN) when models are trained on more upregulated and nonresponsive genes. The percentage of total FNs, for example, was 23.64% for the median performing model trained on all upregulated genes, whereas it was 32.97% when the 250 most responsive upregulated genes were used. Given that F1 is the harmonic mean of precision and recall, F1 increased as well when a model was trained on more upregulated genes. The median F1 varied maximally 0.03 for drought and 0.04 for heat across train sets within test sets (**Figure 2C**). As for the precision, the AU-ROC was roughly similar for models trained on different subsets of upregulated genes. The median AU-ROC varied maximally 0.03 for drought and 0.04 for heat across train sets within test sets (**Figure 2D**). Altogether, these results indicate that it is not necessary to train a model on all available genes. However, training a model on too few upregulated and nonresponsive genes could compromise the recall and cause more FN predictions. Based on these findings, we decided to work with the top 500 and top 1000 upregulated genes, for drought and heat respectively, in future experiments.

After addressing how upregulated gene selection impacts model training and performance, we determined whether the predictive power of a model increases by using information further upstream of the TSS. Promoters span a region both upstream and a short distance downstream of the TSS (Sharon et al., 2012; Redden and Alper, 2015; Srivastava et al., 2018). The core promoter, which typically comprises −40 to +40 relative to the TSS, is a structurally and functionally diverse transcriptional regulatory element from which transcription is initiated by the RNA polymerase II machinery (Juven-Gershon and Kadonaga, 2010; Srivastava et al., 2014). To predict gene expression in response to abiotic stress, we therefore considered 100 bp downstream of the TSS as part of the promoter (see Materials and Methods). Plant promoter architecture is important for understanding the regulation of gene expression in plants. We compared the performance between models trained on proximal (300bp prom, including 100bp 5’UTR) and distal (1000bp prom, including 100bp 5’UTR) TFBSs and pCREs (**Figures 3A–D**). The same five train-test splits as for **Figure 2** where used. No major differences were observed in any of the model performance metrics between models using a distal or a proximal promoter for both heat and drought, respectively. There is a maximum variation of 0.04 across model performance metrics between the proximal and distal promoter. To conclude, our results suggest that when using pCREs and TFBSs, the proximal promoter suffices for gene expression prediction in response to heat or drought. For further experiments, we therefore decided to use the proximal promoter.

Because we use pCREs, together with known TFBSs, to train RF models to discern upregulated from nonresponsive genes, we studied the impact of the applied k-mer finding approach, used to identify pCREs, on model performance and the most important motifs for gene expression prediction. In contrast with previous studies that use homemade scripts for motif discovery (Azodi et al., 2020; Meng et al., 2021; Moore et al., 2022), we compared three different approaches to identify overrepresented oligomers in gene promoters: ROA, ROD and PG (see Materials and Methods). The same five train-test splits as for **Figure 2** where used. In total 643, 364 and 540 significantly overrepresented k-mers were identified by PG, ROD and ROA, respectively (**Figure S3A**). ROD is clearly the more stringent approach, particularly considering that enriched k-mers in nonresponsive compared to upregulated genes are also included therein. ROA identified less 6-mers but substantially more 8-mers compared to ROD. PG identified more 6 and 7-mers than ROD, as well as 9-mers and longer oligomers not detected by ROD and ROA. To assess the impact of k-mer finding strategy on the prediction of gene expression in response to heat or drought, we compared the performance of models trained on pCREs identified using PG, ROA and ROD. Albeit considerable difference in the number and length of detected overrepresented k-mers (**Figure S3A**), there was no major difference, for heat and drought, in the performance of models trained on pCREs identified by each of the three k-mer finding approaches. Model performance metrics varied maximally 0.04 between applied k-mer finding approaches for heat and drought (**Figures 4A–D**). The comparable performance, despite the considerably lower number of overrepresented pCREs detected by ROD, suggests that more redundant and/or less important pCREs are identified using ROA and PG. Subsequently, the highest ranking motifs for the prediction of gene expression in response to heat were compared between RF models trained on TFBSs, and pCREs identified by ROD, ROA and PG (**Figure S3B**). For ROD, 63 of the 364 (17%) enriched pCREs were considered highly important. Of the 540 overrepresented pCREs detected by ROA, 75 (14%) were highly important. For PG, 85 of the 643 (13%) overrepresented pCREs were considered highly important (**Figure S3A**). The longer the k-mers, the smaller their share in the top-ranking pCREs, indicating that longer pCREs contribute less to a correct prediction compared to shorter ones. This finding explains in part why less of the overrepresented pCREs detected by ROA and PG are top-ranking motifs. Among the most contributing pCREs, 29 are in common between ROD and ROA. Both ROD and ROA share 19 and 25 most important pCREs, respectively, with PG (**Figure S3B**). We also compared the main pCREs and their rank between the three k-mer finding approaches (**Figure 4E**). Globally, there are both common and specific pCREs and their rank depends on the used k-mer finding approach. Unique for ROD are the pCREs with a negative SHAP value rank, which represent the pCREs that are enriched in the nonresponsive compared to the upregulated genes (**Figure 4E**, ROD, bottom pCREs). To conclude, the number of overrepresented pCREs and their length varies strongly depending on k-mer finding approach. The majority of most important pCREs for gene expression prediction in response to heat are 6-mers. This explains, in part, the relatively minor impact of the applied k-mer finding approach on model performance, as these differ considerably in the number of k-mers longer than six nucleotides that are detected. Because of the similar performance with other k-mer finding approaches, the relative highest number of most important pCREs for gene expression prediction and the ability to simultaneously compute overrepresented pCREs in upregulated compared to nonresponsive and the other way around, ROD was chosen as the preferred k-mer finding approach for further experiments.

### 3.3 Modeling the temporal transcriptional response to abiotic stress to identify the most important regulatory motifs

The temporal differences in transcriptional reprogramming reported in **Figure 1** indicates that the regulation of gene expression in response to heat or drought changes over time. To determine the most important regulatory motifs for predicting the temporal heat or drought response, four time points were selected. The clustering of time points (**Figures 1A, B**) as well as the enrichment of TFBSs and GO Biological Process terms in upregulated genes in response to heat or drought (**Figures S1, S2**) were used to make an informed selection. Time points 1, 6, 12 and 24h were chosen for heat stress. For these time points, replicates per time point group together well and are secluded from the control (0h) (**Figure 1A**). HSF motifs are strongly enriched at time point 1h, whereas NAC and MYB motifs are not. Consistently, (cellular) response to heat is strongly enriched. At time point 6h, MYB related motifs are overrepresented. MYB-related as well as HSF motifs are enriched at time point 12h. Similar to time point 1h, response to heat is overrepresented in the upregulated genes. Time point 24h is characterized by an enrichment of NAC motifs (**Figure S1**). For drought stress, time points 1, 6, 24 and 36h were selected. Replicates per time point cluster together well and are isolated from the control (0h) (**Figure 1B**). At time points 6 and 24h there is a modest and strong enrichment of MYB related motifs, respectively, whereas time points 1h and 36h are characterized by an enrichment of WRKY motifs. There is some HSF enrichment at time point 1h. Consistently, response to heat is enriched for the latter. Time point 24h is interesting because of the enrichment of circadian rhythm. For none of the four considered time points, however, response to water deprivation or response to dehydration is enriched (**Figure S2**).

**Figure 5:**
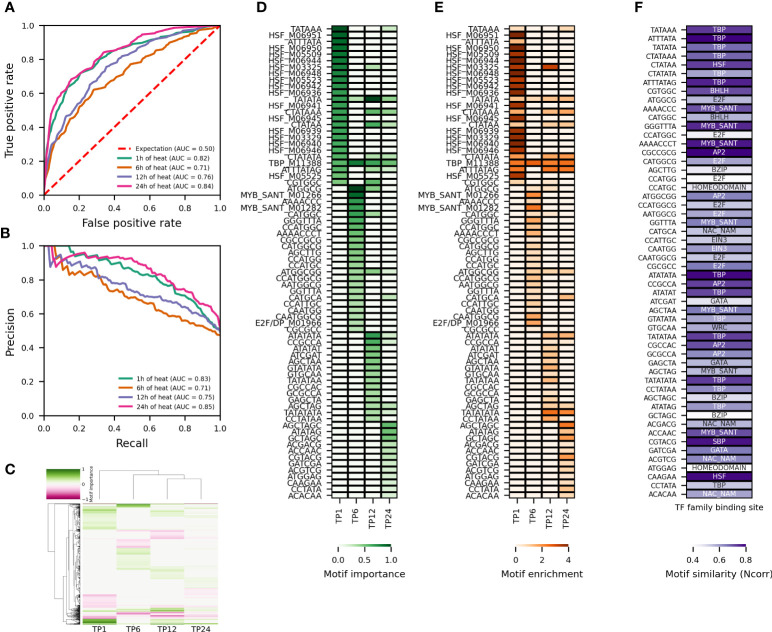
Temporal variation in model performance and most important (putative) regulatory motifs for the response to heat. **(A, B)** Comparison of AU-ROC and AU-PR, respectively, across time points for RF models trained on pCREs and TFBSs contained in the proximal promoter of the top 500 upregulated genes subset. The area under the curve (AUC) is reported for each trained model. **(C)** Hierarchically-clustered heatmap of the most important motifs across time points. Motifs with a positive SHAP importance are indicated in green, those with a negative SHAP importance in pink. **(D)** Heatmap of the motif importance and **(E)** motif enrichment for the 25 most important motifs with positive SHAP value rank across time points. **(F)** Heatmap of the motif similarity (Ncorr) for the pCREs among the 25 most important motifs with positive SHAP value rank across time points.

To study the temporal variation in the transcriptional reprogramming in response to abiotic stress, five new train-test splits were performed for each of the concerned time points. In contrast to **Figures 2**–**4**, now the common nonresponsive genes across the concerned time points were used. A model was trained on each of the five train sets per time point and only the results for the median performing model, based on F1, are shown (**Figures 5**, **S4**). We first compared the model performance to predict gene expression for different time points (**Figures 5A, B** for heat, **Figures S4A, B** for drought). For heat, there is more variation in model performance across time points compared to drought. The AU-ROC and AU-PR range from 0.71-0.84 and from 0.71-0.85, respectively (**Figures 5A, B**). For drought, the AU-ROC and AU-PR range from 0.8-0.86 and from 0.82-0.87, respectively (**Figures S4A, B**). Consistent with the larger variation in model performance, time points differ more clearly in the main motifs for gene expression prediction in response to heat (**Figure 5C**). Time points 12 and 24h group together, whereas 1 and 6h are more different. For drought, consistent with the smaller variation in model performance, time points are more similar with regard to the most important motifs for gene expression prediction (**Figure S4C**). Time points 6 and 36h group together, and 1 and 24h group together. Next, we compared the motif enrichment of the top 25 motifs with positive SHAP value rank (indicative of relevance for upregulated genes), for predicting gene expression in response to abiotic stress (**Figure 5D** for heat, **Figure S4D** for drought). For heat and drought, the majority of most important motifs are pCREs. However, for heat, there are considerably more TFBSs among the highest ranking motifs compared to drought. Consistent with the hierarchical clustering (**Figure 5C**), there is a clear difference in the top 25 motifs between the different time points for heat (**Figure 5D**). The TFBSs are dominated by HSF binding sites, all of which can be traced back to time point 1h. Also a TATA-binding protein (TBP) binding site is part of the highest ranking TFBSs. The most important pCREs for time point 1h are related to TBP, HSF and Basic Helix Loop Helix (bHLH) binding sites (**Figure 5F**). For time point 6h, one TBP, two MYB and one E2F/DP TFBSs are among the 25 highest ranking TFBSs (**Figure 5D**). Consistently, one, two, four and eight of the most important pCREs are related to TBP, AP2, MYB and E2F binding sites, respectively. The other pCREs are similar to bHLH, bZIP (basic leucine zipper) and NAC binding sites, among others (**Figure 5F**). The main TFBSs for time point 12h are one TBP and one HSF (**Figure 5D**). Ten TBP, one HSF, two bHLH, one E2F, four AP2, two GATA and two MYB binding site related pCREs are among the highest ranking motifs. Only one TBP TFBS is part of the most important motifs for gene expression prediction at time point 24h, the others are pCREs. Of the latter, nine are related to TBP, four to NAC, two to HSF, two to bZIP, two to MYB and two to AP2 binding sites, among others. All time points share one TBP TFBS and one TBP associated pCRE. Three time points – 1, 12 and 24h – share three TBP binding site related pCREs and one HSF related pCRE. Two TBP, one MYB and one AP2 bindings site related pCREs are shared between time point 12 and 24h. Overall these results indicate both time point-specific (E2F/DP, bHLH, NAC, bZIP, GATA) and more common regulatory elements (TBP, HSF, MYB).

As opposed to heat, there is more similarity between the 25 highest ranking motifs across time points for drought (**Figure S4D**), which is consistent with the hierarchical clustering of the most important motifs (**Figure S4C**). Across the four time points, the highest ranking motifs are dominated by pCREs. For time point 1h, seven, five, one and three pCREs are related to TBP, MYB, AT-HOOK and GATA bindings sites, respectively (**Figure S4D**). Also NAC, bZIP, E2F and bHLH motifs add to the list of main pCREs. Ten TBP, four MYB, two GATA and three AT-HOOK binding site related pCREs are among the highest ranking motifs for time point 6h. The other pCREs are related to HSF, NAC, and bHLH binding sites. Consistent with the hierarchical clustering of the main motifs (**Figure S4C**), time point 24h is clearly different from the other time points concerning the 25 highest ranking motifs. Only six of the most important pCREs, five related to a TBP and one related to a MYB binding site, are in common with other time points. There are five TFBSs among the highest ranking motifs, all are MYB-related. Interestingly, the MYB binding site and the five MYB-related pCREs, are all potential REVEILLE (REV) binding sites. REV TFs are involved in regulating the plant circadian rhythm (Rawat et al., 2009), suggesting that this biological process affects gene expression at time point 24h. Time point 36h again has a lot of common pCREs with time points 1 and 6h (**Figure S4D**). Nine, four and three pCREs are related to TBP, MYB and GATA binding sites, respectively. The other pCREs are related to bZIP, HSF, bHLH, AT-HOOK and NAC binding sites, among others. Also a TBP TFBS is part of the highest ranking motifs. Overall, these findings indicate more common regulatory elements for drought compared to heat. TBP, MYB, bZIP, AT-HOOK, GATA binding sites emerge as general regulators of the transcriptional response to drought, whereas E2F/DP, bHLH, HSF and NAC are more time point-specific regulatory elements. Furthermore, our results suggest that known TFBSs are less useful for predicting response to drought compared to heat.

To better understand the underlying factors that contribute to the importance (SHAP value rank) of a motif, we investigated the presence of the highest ranking motifs in upregulated compared to nonresponsive genes (**Figure 5E** for heat, **Figure S4E** for drought). From **Figures 5E** and **S4E**, it immediately becomes clear that known TFBSs are present in considerably more upregulated relative to nonresponsive genes compared to pCREs. Their higher prevalence is due to the nucleotide degeneracy inherent to known TFBS PWMs, whereas pCREs do not allow such a degeneracy. However, despite their lower occurrence in upregulated relative to nonresponsive genes, some pCREs have a higher importance for gene expression prediction compared to high ranking TFBSs. For example, TATAAA, related to a TBP binding site, is the highest ranking motif for predicting gene expression in response to 1h of heat (**Figure 5E**). It has a motif fold enrichment of 0.7 and is present in 34.6% of the upregulated genes, but also in 21.5% of the nonresponsive genes used for model training. The known HSF binding site which has the second-highest rank occurs in 16.9% of the upregulated genes but only 0.7% of the nonresponsive genes used for model training. Therefore, its motif fold enrichment of 4.6 is higher than that of the higher ranking TATAAA motif. Hence, the importance of a motif for predicting gene expression in response to abiotic stress cannot be solely ascribed to a strong enrichment in upregulated compared to nonresponsive genes. The same finding is true for the response to drought (**Figure S4E**). SHAP values take into account the interaction between each feature and the other features in the model and thus reflect not only independent effects of a feature on the prediction but also its combined effect with other features (Lundberg et al., 2020). Consequently, co-occuring high-ranking motifs with relatively low motif fold enrichment in the proximal promoter of upregulated genes are important for the prediction of gene expression in response to abiotic stress.

### 3.4 Quantifying the relative importance of coding and noncoding features for predicting the transcriptional response to abiotic stress

**Figure 6:**
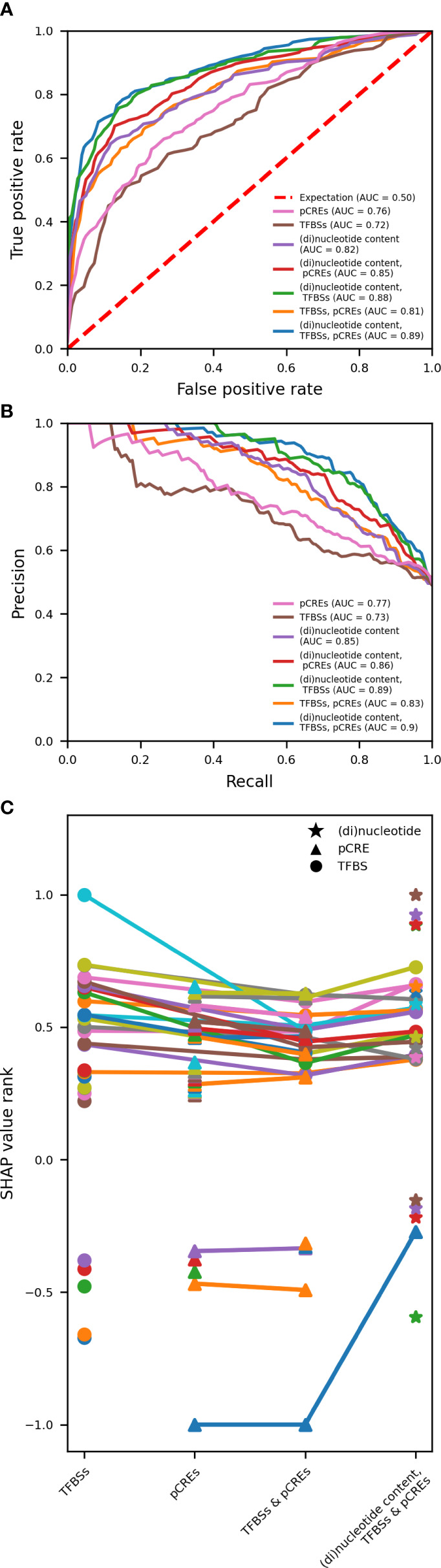
Variation in model performance and most important features for predicting gene expression in response to 1h of heat, based on the used feature space for model training. **(A, B)** Comparison of AU-ROC and AU-PR, respectively, for models trained on pCREs, TFBSs and (di)nucleotide content and all possible combinations. pCREs and TFBSs represent noncoding features in the proximal promoter. (Di)nucleotide content represents both coding and noncoding features in the proximal promoter, open reading frame, and 3’UTR. The area under the curve (AUC) is reported for each trained model. **(C)** The top 25 features with a positive and the top 5 features with a negative SHAP value are compared between models trained on TFBSs, pCREs, TFBSs & pCREs and TFBSs, pCREs and (di)nucleotide content. Common most important features are connected using a line. Different markers are used for the different types of features.

We have shown that gene expression in response to abiotic stress can be predicted based on known and putative regulatory motifs in the promoter of a gene. Previous research indicated that gene expression regulation spans both coding and noncoding regions of a gene (Washburn et al., 2019; Zrimec et al., 2020; Zrimec et al., 2022). Meng and collaborators have demonstrated that the transcriptional response to cold stress can be accurately predicted using the nucleotide and dinucleotide content of various genomic regions, covering both coding and noncoding DNA (Meng et al., 2021). To study the importance of pCREs, TFBSs and (di)nucleotide content for gene expression in response to abiotic stress and to assess whether coding features can further improve model performance when combined with noncoding features, we trained RF models on pCREs, TFBSs and the (di)nucleotide content of coding and noncoding regions, and all possible combinations (**Figures 6** and **S5**).

For drought and heat, we used to one time point to study the effect of using different types of features on model performance. Rice was shown to exhibit a fast response to heat and a slow response to drought (Wilkins O. et al., 2016). At 1h of heat, HSF motifs and response to heat are strongly enriched in upregulated genes (**Figures S1A, B**). At 6h of drought, upregulated genes are characterized by an enrichment of MYB motifs and cellular redox homeostasis (hydrogen peroxide metabolism, response to oxidative stress, cellular oxidant detoxification) (**Figures S2A, B**). Therefore, time point 1h was selected for heat and 6h for drought. The same five train-test splits per time point as for **Figures 5**, **S4**, were used for model training and testing. There is a pronounced variation in the performance of a model to predict gene expression in response to 1h of heat based on the features included for model training (**Figures 6A, B**). The performance is the lowest (AU-ROC of 0.72 and AU-PR of 0.73) when a model is trained on TFBSs alone, whereas it is the highest (AU-ROC of 0.89 and AU-PR of 0.9) when trained on pCREs, TFBSs and (di)nucleotide content. Comparing the latter to a model trained on pCREs and TFBSs (AU-ROC of 0.81 and AU-PR of 0.83), suggests that the (di)nucleotide content further adds to a correct prediction of the transcriptional response to heat. Moreover, training a model on just (di)nucleotide content of coding and noncoding DNA yields a better performance than models trained on pCREs or TFBSs. Compared to heat, the variation in model performance to predict gene expression in response to 6h of drought is smaller (**Figures S5A, B**). The performance of a model trained solely on TFBSs is lower compared to a model trained on other (combinations of) features. The performance is lowest for the latter (AU-ROC of 0.72 and AU-PR of 0.71) and the highest for models trained on pCREs and (di)nucleotide content (AU-ROC of 0.88 and AU-PR of 0.91). There is no further increase in model performance, when TFBSs are also used for training. These results suggest that known TFBSs are inferior to pCREs for the prediction of the transcriptional response to 6h of drought. Nevertheless, the performance of models trained on pCREs and TFBSs is 1% higher than that of models trained solely on pCREs.

To get a better understanding of the relative contribution of different features (known and putative regulatory motifs, noncoding and coding features) to the prediction of gene expression in response to heat or drought, the 25 highest ranking positive features and the five highest ranking negative features were compared when models are trained on: 1) TFBSs, 2) pCREs, 3) pCREs & TFBSs and 4) pCREs, TFBSs & (di)nucleotide content (**Figures 6C**, **S5C**, and **Table S1**). For the response to 1h of heat, 21 of the 25 highest ranking positive TFBSs are HSF binding sites when trained on TFBSs alone. The remaining four are two TBP binding sites, one TALE and one NAC binding site. Consistently, when only pCREs are used for model training, of the 25 positive pCREs, eight are related to TBP and ten to HSF binding sites. The remainder are related to GATA, EIN3, AP2, E2F/DP and bHLH binding sites. If a model is trained on pCREs and TFBSs, 16 of the 25 highest ranking positive motifs are known HSF binding sites, while one is a known TBP binding site. All of these 17 binding sites are in common with those when a model is trained solely on TFBSs. The other 8 are pCREs: ATATAG (TBP), CTATATA (TBP), ATTTATAG (TBP), CTATAA (HSF), TATAAA (TBP), CTATAAA (TBP), TATATA (TBP) and ATTTATA (TBP). Thus, whereas, nine pCREs related to an HSF binding site are part of the top 25 motifs for a model trained on pCREs, only one remains when a model is trained on pCREs and TFBSs. These results suggest that when both known and putative HSF binding sites are used for model training, known HSF binding sites have greater added value. Because of their strongly negative importance, motifs GGCCCA (TCP), CTCCCC (TCP), CCCAAA (MADS-BOX), ATGGGCC (TCP) and ACCCTA (MYB) appear to be characteristic of unresponsive genes to heat stress. If trained on pCREs, TFBSs and (di)nucleotide content, 15 of the 25 highest ranking features are known HSF binding sites, which are in common with those when a model is trained on TFBSs and TFBSs & pCREs. The other ten are all CDS (di)nucleotides. The nucleotide content of GC, AT, CG and G in the coding sequence are the highest ranking features. These results indicate that the (di)nucleotide content of the coding sequence can further improve model performance.

In sharp contrast with heat, only one TBP binding site has a high motif importance relative to the other 24 highest ranking TFBSs for predicting the transcriptional response to drought, including WOX, SET, bZIP, E2F/DP, HD-ZIP, C2H2, G2-like, HSF, NAC and various MYB and MYB-related bindings sites (**Figure S5C** and **Table S1**). This could explain the lower model performance of models trained on TFBSs alone (**Figures S5A, B**). When a model is trained on pCREs and TFBSs, the TBP binding site showing high motif importance if trained on TFBSs alone, is part of the highest ranking motifs, however, its relative importance is considerably reduced when a model is trained on pCREs as well. The remaining 24 top motifs are thus pCREs, 22 of which are in common with the highest ranking pCREs when a model is trained solely on pCREs. Of these, nine have similarity with TBP binding sites, three with MYB, three with GATA, three with AT-HOOK and two with bZIP. The pCREs CGATCG (GATA), AGCTAC (MYB) and ATATAG (TBP) are specific for the model trained on pCREs alone whereas CGATCA (GATA) and ATATATA (TBP) are specific for the model trained on pCREs and TFBSs. Because of their strongly negative importance, motifs GGCGGA (AP2), GAGGAGA (SBP), CTCCGC (AP2), CGGCGGA (AP2) and CCCCTC (TCP) appear to be characteristic of unresponsive genes to drought stress. When a model is trained on pCREs, TFBSs and (di)nucleotide content, no TFBSs are among the 25 highest ranking features. Eight of these 25 features are pCREs: ATTAAT (AT-HOOK), AGCTAG (MYB), TATAAATA (TBP), TAGCTA (MYB), TATAAA (TBP), CATGCA (NAC), TAATTAA (HOMEODOMAIN) and AATTAA (AT-HOOK). The remaining 17 most important features are (di)nucleotides, four are intron and 13 are coding sequence (di)nucleotides. The content of CG, AT, AC, and TT in the codings sequence constitute the highest ranking features. Hence, similar to heat, the (di)nucleotide content of the coding sequence can further improve the performance of a model to predict the transcriptional response.

## 4 Discussion

Global warming causes aggravated heatwaves and devastating droughts, posing a serious threat to our food production. We are in need of crops that can produce more with less input and are resilient to unfavorable growth conditions. To purposefully engineer plants, further deciphering of the regulatory grammar driving gene expression in response to abiotic stress is indispensable. Supervised machine learning has enabled significant improvements in the prediction of transcriptional responses and has proven to be a powerful and versatile tool to identify important regulatory elements. Optimizing training data and tuning machine learning models is a challenging and iterative process that depends on the goal of modeling. More training data generally improves the prediction performance. In this study, however, the goal is not to forecast stress responsive genes, but to identify the major drivers of gene expression prediction to unravel the regulatory grammar of the transcriptional response to abiotic stress. For that purpose, using more genes could be inferior to using more informative genes. Our results suggest that it is not necessary to use all available genes. An equally good performance can be achieved for both heat and drought by training an RF model on a well-considered selection of the most and least responsive genes, and has some major advantages compared to using the complete gene set for model training. Building a feature space and training a model is computationally less expensive and time-consuming. Using the same number of genes across time points allows a more reliable comparison of model performance, which is important considering the temporal variation in the number of differentially expressed genes in response to abiotic stress. The computational cost of calculating SHAP values, used as a measure of relative feature importance, increases with the number of features and the depth of decision trees. When calculating the SHAP values for an RF model trained on all nonresponsive and upregulated genes in response to heat (> 4000 genes), it is required to calculate SHAP values on a sample of genes, whereas this was not the case for the 1000 most upregulated genes subset. Computing SHAP values based on a sample of genes reduces the reliability of the importance estimate. Hence, by working with a well-considered selection of genes (for heat the 1000 most responsive upregulated genes), the calculation of the feature importances is based on the same number of genes as used for model training.

Gene expression is mainly, but not exclusively, regulated at the level of the promoter – the cis-regulatory region flanking the TSS. Most binding sites of TFs and other RNA-polymerase related proteins controlling transcription initiation, occur upstream of the TSS (de los Reyes et al., 2015). The distance of TF binding sites relative to the TSS varies substantially across genes. The promoter’s regulatory architecture is key to understand gene expression regulation. Using proximal or distal promoter regulatory elements for model training revealed, for both heat and drought, a comparable performance, indicating that the upstream region closest to the TSS is the most important for gene expression prediction. In agreement with our findings, the performance to classify maize nonresponsive and upregulated genes in response to heat was similar for models trained on the cis-regulatory information in the 500 bp compared to the 2 kb region upstream of the TSS (Zhou et al., 2022).

To unravel the regulatory grammar controlling the transcriptional response to abiotic stress by identifying major drivers of gene expression, we trained ML models on both known and putative regulatory elements. The most important motifs for gene expression prediction are interesting for the design of synthetic stress-inducible promoters to overcome shortcomings of native promoters in crop engineering (Mehrotra et al., 2011; Liu and Stewart, 2015; Rushton, 2016). TFBSs can be collected from existing databases, whereas pCREs are identified by determining enriched oligomers in upregulated compared to nonresponsive genes, potentially yielding novel regulatory motifs. We demonstrate that there is a minor impact of k-mer finding approach on model performance and the most important features for gene expression prediction in response to abiotic stress. However, we have identified several advantages and disadvantages associated with their usage. ROD can simultaneously identify k-mers enriched in upregulated compared to nonresponsive genes, and the other way around. For the purpose of designing synthetic stress-inducible promoters, this is a valuable asset, as those characteristic of nonresponsive genes should be excluded from the design. ROA uses a custom background model, containing the frequencies of 6, 7 and 8-mers in nonresponsive genes, instead of the actual promoter sequences for the enrichment analysis. Both ROA and ROD cannot detect oligomers longer than 8 nucleotides, however, given that the most important pCREs for predicting the response to heat or drought are predominantly 6 and 7-mers, this does not seem to be a major shortcoming. For each oligomer of length k, both approaches identify overrepresented k-mers independently of shorter and longer ones, resulting in redundancy among significantly enriched k-mers. The PG strategy keeps only significantly overrepresented k-mers that have a lower p-value than its predecessor, reducing the redundancy among significantly enriched k-mers. However, overrepresented k-mers that have no shorter, significantly enriched variant are not identified.

Temporal discrepancies in transcriptional reprogramming imply variation in the regulatory grammar driving gene expression over time. We therefore modeled the transcriptional response to heat or drought for different time points and compared the most important regulatory motifs. For heat, there is a pronounced temporal variation in model performance and most important motifs for gene expression prediction. Consistent with previous studies, our results suggest that HSF and TBP regulatory elements are key for the early response to heat. HSFs were shown to activate the expression of heat shock and other heat responsive genes by binding to promoter Heat Shock Elements (HSEs) (Guo et al., 2016). A synthetic promoter, consisting of an HSE upstream of a TATA-box, was capable of driving the expression of a GUS gene in response to heat in multiple plant species, including rice (Maruyama et al., 2017). Furthermore, HSF1, a central regulator of the heat stress response, was shown to directly interact with TBPs, the general TATA-box binding TFs (Reindl and Schöffl, 1998; Savinkova et al., 2023). At later time points, regulatory elements associated with MYB, E2F/DP, AP2, NAC, bHLH and bZIP TFs were the most important for predicting the transcriptional response to heat. Consistently, various genes encoding HSF, MYB, bHLH, E2F/DP, NAC, bZIP TFs were upregulated in response to heat at some point in time. Previous research established a role for MYB, AP2, NAC and bZIP TFs in the regulation of heat-responsive genes (Xie et al., 2019; Zhao et al., 2020; Park et al., 2021). Some bHLH genes were shown to exhibit altered expression in response to heat (Zhang et al., 2018; Zhang et al., 2020) and a bHLH was identified in hybrid rice under heat stress (Wang Y. et al., 2020). Vandepoele and collaborators identified various E2F target genes in rice, involved in cell cycle regulation and DNA replication (Vandepoele et al., 2005). In *Arabidopsis*, it was shown that the function of E2F/DP proteins is mainly controlled by their nuclear localization, mediated by their interaction with other proteins (Kosugi and Ohashi, 2002). Heat stress is known to pause cell cycle progression (Eekhout and De Veylder, 2019). Our identification of E2F/DP related regulatory elements as important motifs for the gene expression prediction at this time point, thus suggests a role for E2F/DP TFs and their target genes in the response to heat. Consistently, an E2F/DP binding site emerged as a highly ranked motif for predicting the transcriptional response to combined heat and drought stress in *Arabidopsis* (Azodi et al., 2020). TBP related regulatory elements are important drivers of gene expression prediction over time. In accordance with the enrichment of MYB related TFBSs in upregulated genes, we show that motifs associated with MYB binding sites are important for the response to heat at later time points (6, 12 and 24h). For drought, there is less temporal variation in model performance and the most important regulatory elements for gene expression prediction over time. More common regulatory elements – TBP, MYB, bZIP, GATA and AT-HOOK – emerge for the response to drought, with time point 24h being the exception. Remarkably, REVEILLE regulatory elements (5′-AAATATCT-3′), constitute the most important motifs for gene expression prediction at this time point, suggesting circadian effects (Rawat et al., 2009). Accordingly, circadian rhythm is enriched in upregulated genes at time point 24h. Similar to heat, TBP and MYB associated regulatory elements are important for predicting the transcriptional response to drought over time. Consistent with previous studies (Zhao et al., 2020; Guo et al., 2021; Hu et al., 2022), also bZIP, bHLH and NAC related motifs emerge as common drivers of gene expression prediction for heat and drought at some point in time. Motifs related to MYB and NAC were also key features for the prediction of the transcriptional response to both heat and drought in *Arabidopsis* (Azodi et al., 2020). GATA regulatory elements play a more prominent role in predicting the transcriptional response to drought compared to heat. In rice, OsGATA8 was reported to regulate the expression of key genes involved in drought tolerance and the scavenging of ROS (Nutan et al., 2020). In tomato, overexpression of SlGATA17 was shown to promote drought tolerance (Zhao et al., 2021). AT-HOOK regulatory elements emerge as important drought-specific gene expression predictors. The rice gene OsAHL1, containing an AT-hook motif, was shown to improve drought tolerance in rice (Zhou et al., 2016). In line with our findings, various genes encoding MYB, NAC, bHLH, HSF, bZIP and GATA TFs were upregulated in response to drought at some point in time.

Previous studies have shown that known and putative regulatory elements differ in their relevance for gene expression prediction (Azodi et al., 2020; Moore et al., 2022). To assess their relative importance, we compared the performance and most important motifs between models trained on pCREs and/or TFBSs. For both heat and drought, pCREs outperform TFBSs in predicting gene expression. This was also true for the response to wounding and combined heat and drought stress (Azodi et al., 2020; Moore et al., 2022). For drought, a model trained on TFBSs alone has a poorer performance and training a model on both pCREs and TFBSs fails to improve model performance compared to pCREs alone, indicating that known TF bindings sites are insufficient for predicting the drought response. Consistent with previous results, the most important regulatory elements were related to GATA, AT-HOOK, NAC, TBP, MYB and bZIP. As opposed to drought, the comparable performance of a model trained on TFBSs or pCREs and the improved performance when a model is trained on both, indicate that known TFBSs are valuable for predicting the response to heat and that pCREs and TFBSs are not fully redundant. Consistent with previous results, HSF and TBP related regulatory elements are the most important for predicting the early transcriptional response to heat when a model is trained on pCREs and/or TFBSs. Interestingly, the HSE (5′-GAAnnTTC-3′) does not emerge among the main regulatory elements when a model is trained on pCREs alone, but parts of the consensus HSF binding site do. This is probably due to the presence of degenerate nucleotides in the binding site that are not well modeled using the applied pCRE detection method.

There is compelling evidence that gene expression regulation is not restricted to TFs and the region around the TSS. Also coding regions can be used to predict gene expression (Washburn et al., 2019; Zrimec et al., 2020; Meng et al., 2021; Zrimec et al., 2021). We therefore evaluated the importance of the (di)nucleotide content of both coding and noncoding genomic regions for predicting the transcriptional response to heat or drought. For both heat and drought, training models on (di)nucleotide content outperformed models trained on pCREs and/or TFBSs and further increased the performance when used for training together with pCREs and TFBSs. For both abiotic stresses, the highest ranking features cover multiple (di)nucleotide content features. Consistent with the response to cold stress (Meng et al., 2021), coding sequence (di)nucleotides, in particular CG and AT, emerged as the most important for gene expression prediction in response to heat or drought. A high CG content and a low AT content (data not shown) are associated with a high feature importance, indicating that they are characteristic of upregulated genes. Cytosines within GC sites play a role in regulating gene expression through both methylation-dependent and methylation-independent mechanisms (Hartl et al., 2019; Schmitz et al., 2019). AU-rich elements have been shown to play a role in RNA stability and degradation (Schoenberg and Maquat, 2012). Furthermore, it was shown that the higher the expression of a gene in response to drought, the higher the GC/AT ratio (Mohasses et al., 2020). Hence, (di)nucleotide content proves to be a valuable predictor of gene expression in plants and contributes to the growing awareness that gene expression regulation spans both coding and noncoding regions.

With this study, we present a detailed guide for generating training data and building a feature space based on coding and noncoding features to model the transcriptional response to abiotic stress. Our approach to using machine learning for gene expression prediction is aimed at maximizing the contrast between upregulated and nonresponsive genes by non-random class balancing and using only the most and least responsive genes for model training. Furthermore, we explored the most relevant information for building a feature space (promoter length and k-mer finding approach). We developed a comprehensive methodology for building and interpreting machine learning models that enabled us to identify both time point-specific and common noncoding regulatory elements for the response to heat or drought stress, as well as abiotic stress-specific and common noncoding regulatory elements. We show that the coding sequence (di)nucleotide content can further improve model performance.

## Data availability statement

Publicly available datasets were analyzed in this study. This data can be found here: https://www.ncbi.nlm.nih.gov/Traces/study/?acc=PRJNA530826.

## Author contributions

First authorship: DS and HO share first authorship. Last authorship: KV is last and corresponding author. DS and HO wrote the Introduction and Materials and Methods. DS wrote the Results and Discussion. DS, HO and KV developed the experimental design. HO performed the transcriptome data collection, differential expression analysis, gene family clustering and enrichment analysis of TFBSs and Gene Ontology. DS performed the binning and undersampling of upregulated and nonresponsive genes for class balancing, feature space building, gene-family-guided train-test splits, random forest classifier training, validation, testing and evaluation, and feature importance estimation. All authors contributed to the article and approved the submitted version.
