# Supplementary material for: Predicting transcriptional responses to heat and drought stress from genomic features using a machine learning approach in rice
Source: Front Plant Sci. 2023 Jul 17;14:1212073. doi: 10.3389/fpls.2023.1212073 (PMC10390317; doi:10.3389/fpls.2023.1212073)
Supplement: Supplementary file 1 [file DataSheet_1.zip › Supplementary Figures (2).PDF]

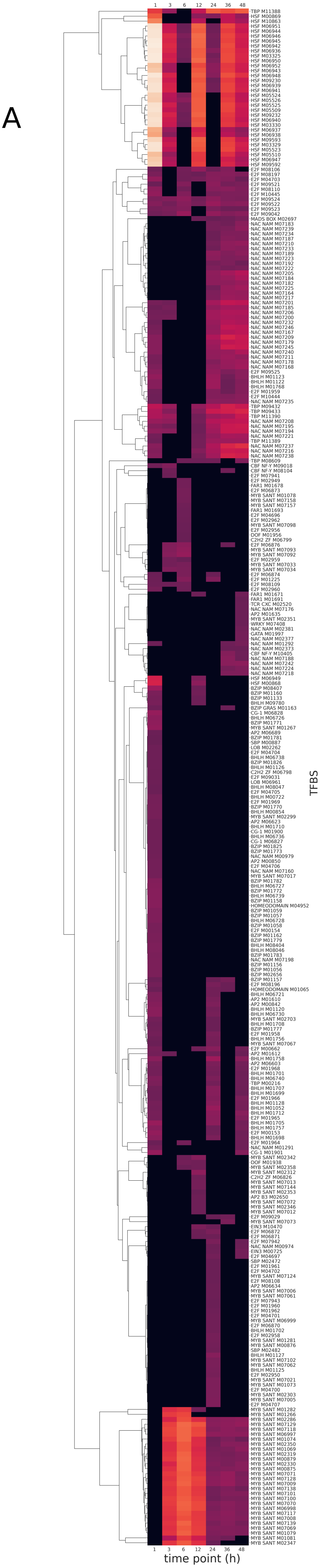

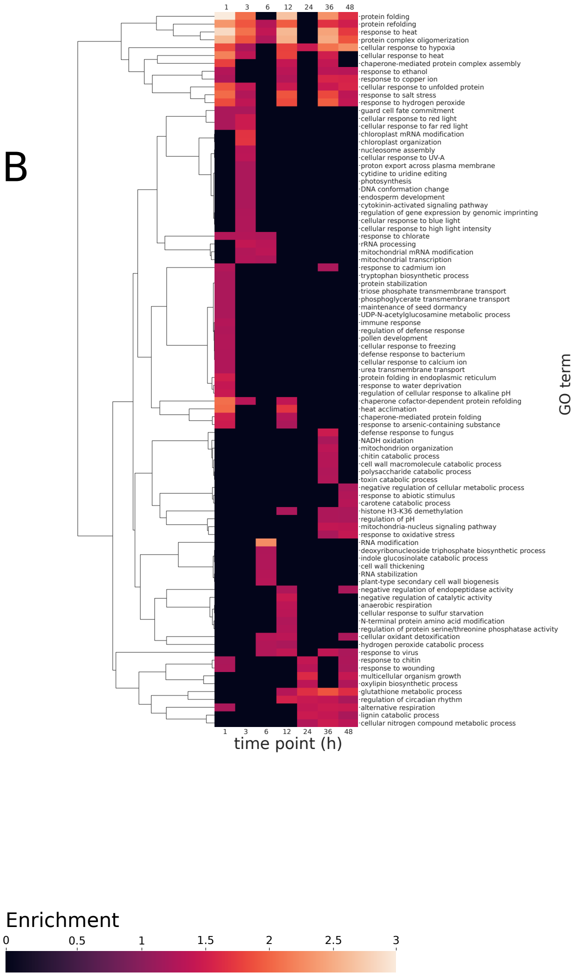

Figure S1 | TFBS and GO term enrichment in heat upregulated genes. (A-B) Enrichment is expressed as the -log10(q-value)^3 of the enrichment fold. **(A)** Enrichment of TFBSs present in the promoters of genes upregulated under heat stress versus nonresponsive genes. The CIS-BP TFBS identifier is annotated with the TF family. **(B)** Enrichment of biological process GO terms, associated with genes upregulated under heat stress versus nonresponsive genes.

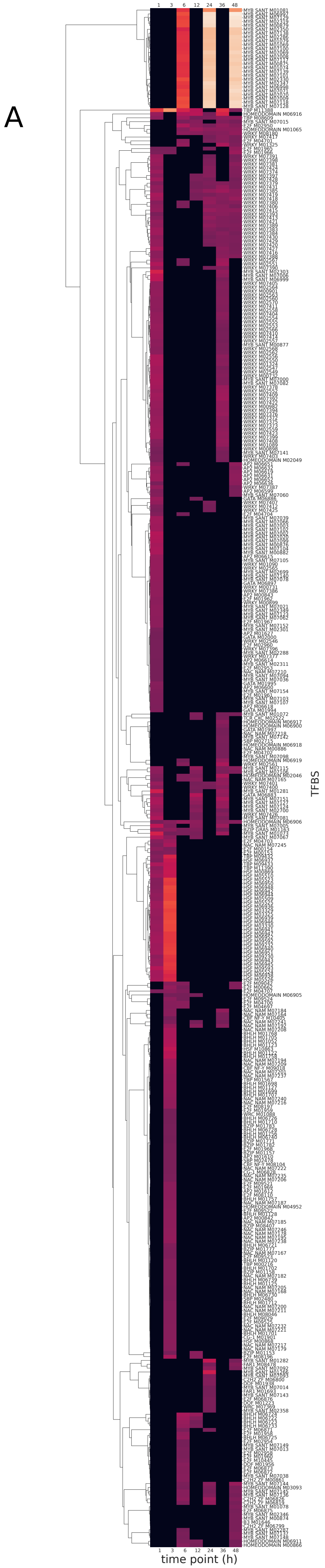

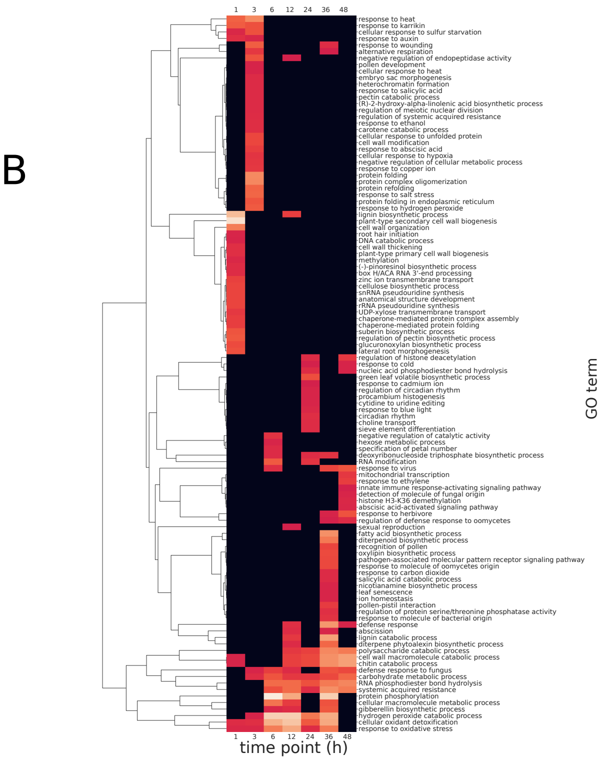

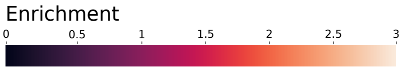

Figure S2 | TFBS and GO term enrichment in drought upregulated genes. (A-B) Enrichment is expressed as the -log10(q-value)^3 of the enrichment fold. **(A)** Enrichment of TFBSs present in the promoters of genes upregulated under drought stress versus nonresponsive genes. The CIS-BP TFBS identifier is annotated with the TF family. **(B)** Enrichment of biological process GO terms, associated with genes upregulated under drought stress versus nonresponsive genes.

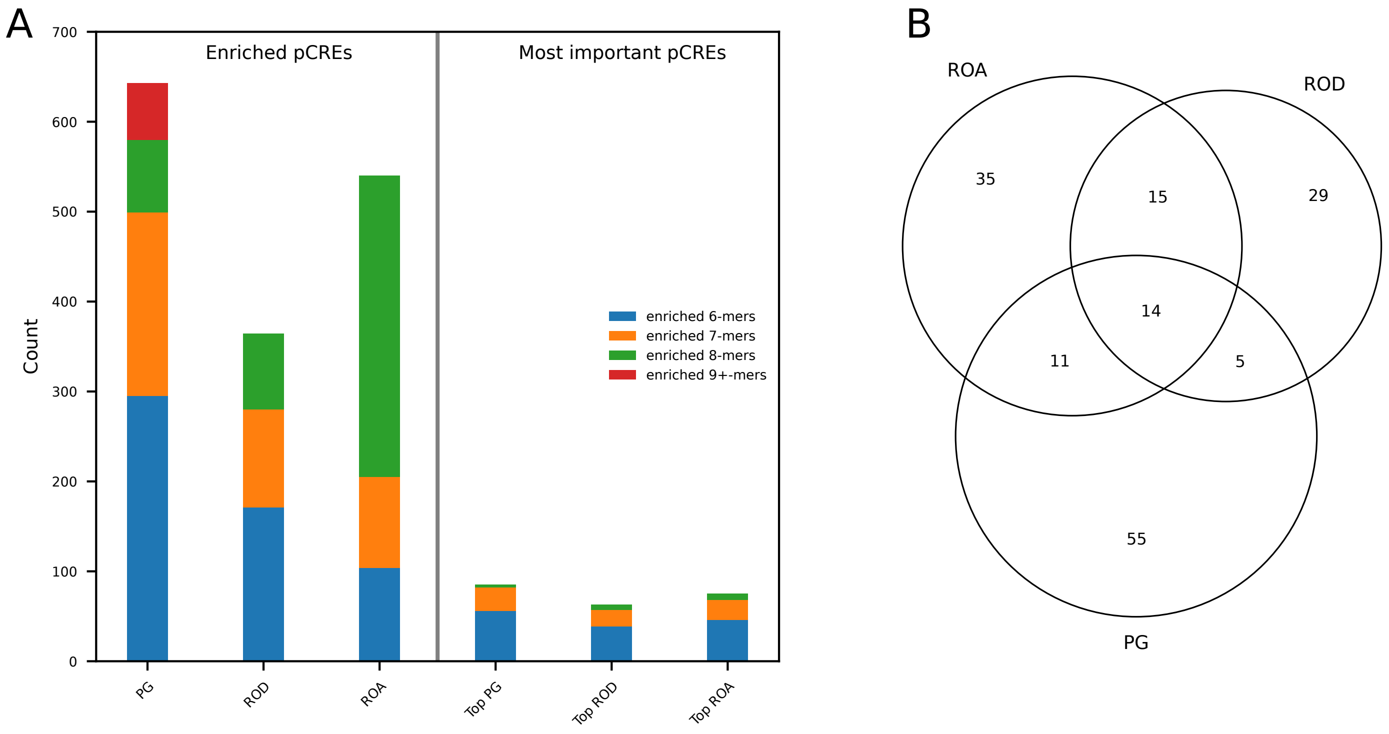

Figure S3 | Overrepresented pCREs and their share in the most important motifs for predicting the transcriptional response to heat based on different k-mer finding approaches. (A) The total number of enriched pCREs in the proximal promoter of the 1000 most strongly upregulated genes in response to 1h of heat using a progressive k-mer growing strategy (PG), RSAT oligo-diff (ROD) and RSAT oligo-analysis (ROA) and the number of oligomers of different lengths is shown on the left. The number of most important pCREs based on SHAP value rank for gene expression prediction and the number of oligomers of different lengths is shown on the right. Different k-mer lengths are represented by different colors. **(B)** The overlap between the most important pCREs, from different k-mer finding approaches, for gene expression prediction.

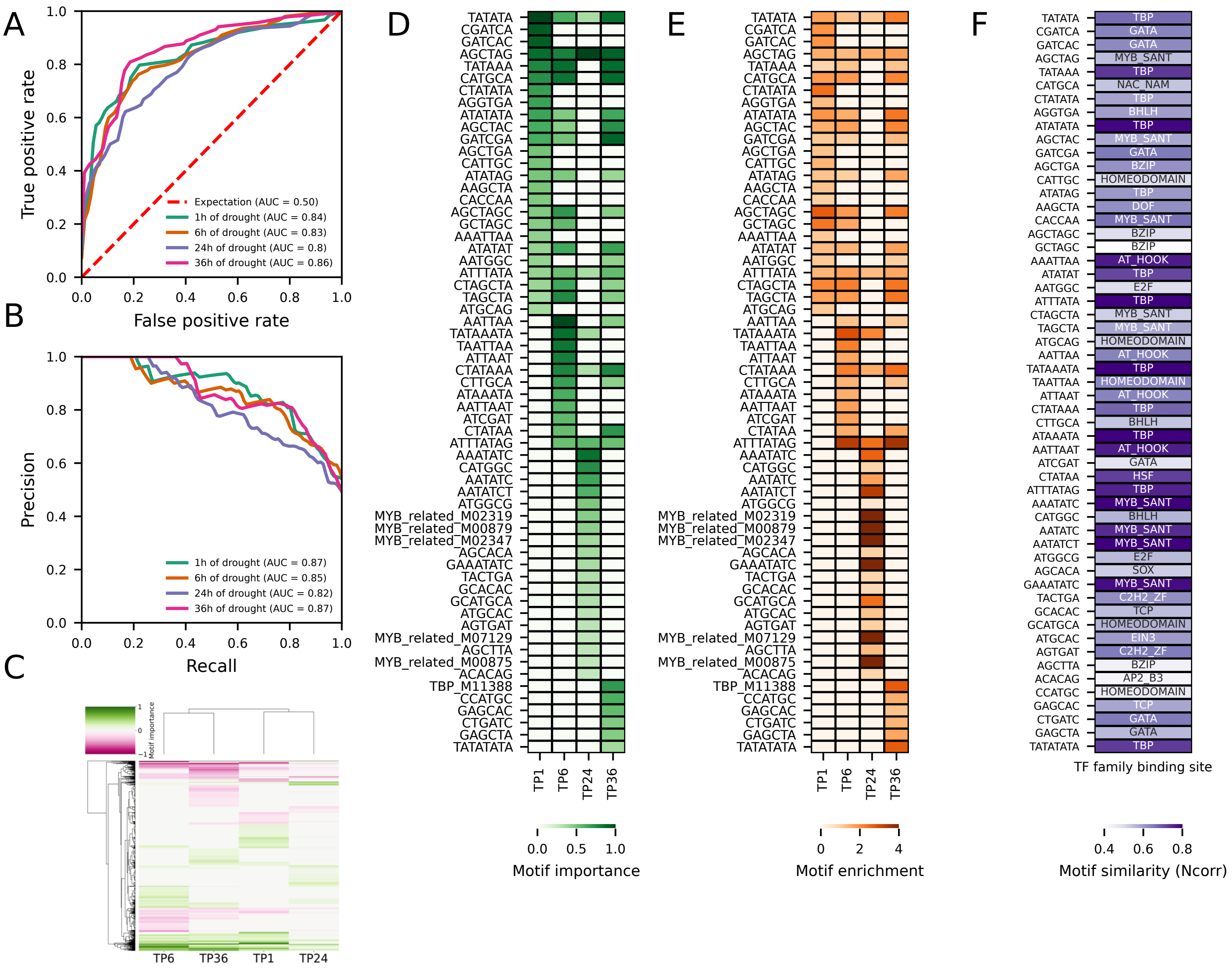

Figure S4 | Temporal variation in model performance and most important (putative) regulatory motifs for the response to drought. (A-B) Comparison of AU-ROC and AU-PR, respectively, across time points for RF models trained on pCREs and TFBSs contained in the proximal promoter of the top1000 upregulated genes subset. The area under the curve (AUC) is reported for each trained model. **(C)** Hierarchically-clustered heatmap of the most important motifs across time points. Motifs with a positive SHAP importance are indicated in green, those with a negative SHAP importance in pink. **(D)** Heatmap of the 25 most important motifs with positive SHAP value rank across time points. **(E)** Heatmap of the motif enrichment for the 25 most important motifs with positive SHAP value rank across time points. **(F)** Heatmap of the motif similarity (Ncorr) for the pCREs among the 25 most important motifs with positive SHAP value rank across time points.

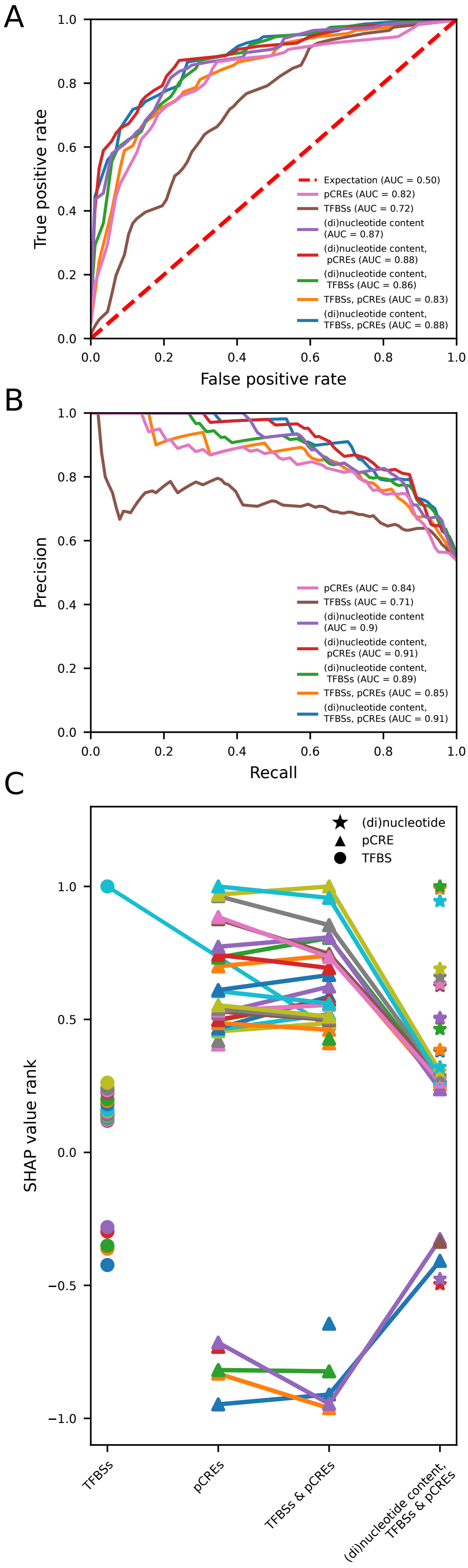

Figure S5 | Variation in model performance and most important features for predicting gene expression in response to drought, based on the used feature space for model training. (A-B) Comparison of AU-ROC and PR-ROC, respectively, for models trained on pCREs, TFBSs and (di)nucleotide content and all possible combinations. pCREs and TFBSs represent noncoding features in the proximal promoter. (Di)nucleotide content represents both coding and noncoding features in the proximal promoter, open reading frame, and 3'UTR. The area under the curve (AUC) is reported for each trained model. **(C)** The 25 most important features with a positive SHAP value and the 5 most important features with a negative SHAP value are compared between models trained on TFBSs, pCREs, TFBSs & pCREs and TFBSs, pCREs and (di)nucleotide content. Common most important features are connected using a line. Different markers are used for the different types of features.
